# Parental Working Hours and Children’s Sedentary Time: A Cross-sectional Analysis of the J-SHINE

**DOI:** 10.2188/jea.JE20200170

**Published:** 2022-01-05

**Authors:** Naoko Hatakeyama, Masamitsu Kamada, Naoki Kondo

**Affiliations:** 1 Department of Health Education and Health Sociology, School of Public Health, Graduate School of Medicine, The University of Tokyo, Tokyo, Japan; 2 Department of Health and Social Behavior, School of Public Health, Graduate School of Medicine, The University of Tokyo, Tokyo, Japan

**Keywords:** sitting time, adolescents, determinants

## Abstract

**Background:**

Sedentary behaviors are prevalent among children and can have a detrimental effect on their health. Little is known about the influence of parental time on children’s sedentary behavior. This study examined the association between parental working hours and children’s sedentary time.

**Methods:**

Cross-sectional data were drawn from the Japanese Study on Stratification, Health, Income, and Neighborhood (J-SHINE) in 2010 and 2011. Participants were 886 children aged 7–18 years and their parents. The primary outcome was self-reported sedentary time after school that comprised screen time and non-screen time. The main explanatory variable was parental working hours. We used multiple regression analysis adjusting for sociodemographic factors.

**Results:**

Children’s mean sedentary time was 222 (standard deviation [SD], 123) min/day; 144 (SD, 108) min/day screen time and 78 (SD, 65) min/day non-screen time. Children whose mothers worked ≥20 hours/week had 28 (95% CI, 9 to 48) min/day longer sedentary time than children of homemakers (240 min/day vs 214 min/day). The longer maternal working hours, the longer sedentary time (*P* for trend <0.01). In contrast, children whose fathers worked ≥48 hours/week had 82 (95% CI, −156 to −7) min/day shorter sedentary time than children of non-working fathers (179 min/day vs 264 min/day). When limited to children whose fathers worked, there was no statistically significant association between children’s sedentary time and paternal working hours.

**Conclusions:**

Children with mothers who work long hours or fathers not working tend to sit more. Supplementing the shortages in resources for childcare may be necessary among those families.

## INTRODUCTION

Sedentary behavior and prolonged sitting time are highly prevalent among children globally.^1^^,^^2^ A study showed that average sedentary time was about 8 to 10 hours/day among children worldwide.^2^ There is a growing body of evidence that children’s sedentary behavior is a risk factor of adverse health outcomes and psychosocial problems, such as being overweight,^3^^,^^4^ suffering from depression,^5^ and sleep problems.^6^ Sedentary behavior, especially screen-based sedentary behavior, such as TV viewing, persist over time in an individual’s lifecourse.^7^^,^^8^ The detrimental health impact of long sedentary time in adulthood includes high risk of all-cause mortality,^9^^,^^10^ cardiovascular disease,^10^ and type 2 diabetes incidence,^10^ independent of physical inactivity. Although there are guidelines for screen time available in some countries, many children do not meet the suggested daily recommendations (eg, ≤2 hours per day in Australia^11^ and Japan^12^). Hence, to develop an effective strategy to reduce children’s prolonged sitting time, it is crucial to investigate the factors that influence it. Several studies have suggested that the potential determinants of prolonged screen time and other sedentary activities include increased popularity of media and internet use,^13^^–^^15^ family-related factors (eg, rule-setting for TV viewing,^16^^,^^17^ TV in children’s bedroom),^18^^,^^19^ and parental lifestyle (eg, parents’ screen-based behavior).^17^^–^^19^

Shortages in resources for childcare among parents are specifically important potential determinants of child screen time as the matter of equity. Childcare requires financial, temporal, and social resources. Study suggests the link of poor household socioeconomic status (eg, low maternal education and low household income) and child screen time.^2^^,^^8^^,^^19^^–^^21^ In Japan, the number of dual-earning couples has nearly doubled from approximately 6 million households in 1980 to 11 million in 2016.^22^ The rate of working mothers in Japan with elementary school children is 75%, and about 80% of families with children are nuclear families,^23^ considered to have very little support for childcare from relatives. Given the increasing working hours and reduced childcare support among families/relatives, strengthening childcare supports in the community and wider society is necessary. However, the current supports may not have sufficiently met the actual needs of parents.^24^ Working mothers have been found to reduce the amount of time spent on childcare due to less time at home.^25^ Some studies indicate maternal long working hours was associated with children’s extended screen time.^26^^–^^28^ In the Japanese metropolitan area, approximately 20% of children 10–12 years old reported that they spent every weekday inside the house.^29^ Though there has been the trend of increasing dual-earning couples worldwide,^30^^–^^32^ dual-working child caring couples might have the sense of support less and actual difficulties if conservative social norms related to child care exist; the national survey showed that nearly half of people agreed with the traditional norm of “male breadwinner and female homemaker” yet in Japan.^33^

However, currently, no research has examined the association between paternal working hours and children’s sedentary behaviors. Moreover, TV viewing and screen time are not a single complete index of sedentary behavior.^34^^,^^35^ To the best of our knowledge, little empirical research has investigated the association of both paternal and maternal working hours and children’s total sedentary time. Therefore, this study aimed to investigate whether paternal and maternal working hours were associated with children’s sedentary time. We also examined this association according to type of sedentary behavior, namely screen-based and non-screen-based behavior, to understand the differences of mechanisms underlying the association between parental working hours and type of sedentary behavior.

## METHODS

### Data

Cross-sectional data were drawn from the Japanese Study on Stratification, Health, Income, and Neighborhood (J-SHINE), which has been described in detail elsewhere.^36^ We used the first wave of the J-SHINE data collected in four municipalities in the greater Tokyo metropolitan area. This dataset was created by linking the data from the survey for an adult family member in 2010, the survey for their spouses in 2011, and the survey for their children in 2011, using unique identification numbers for each household. Participants of the survey for adults ranged between 25 and 50 years old, and those of the child survey ranged between 7 and 18 years old. Adult participants were randomly selected from voter registration lists. Surveyed variables included socioeconomic conditions, health-related behavior, and health status. The exclusion criteria for our analysis included surveys with missing values among outcome or main explanatory variables and not having answered the survey questions by themselves. There were 2,244 families eligible for the child survey of J-SHINE, and 1,520 families had valid data (valid response rate: 67.7%). Among 1,515 children aged 7–18 among the respondent families, our final sample used for analysis consisted of data from 886 children (mean age, 11.9; standard deviation [SD], 3.3 years; boys comprised 53.4% of the children cohort), 579 fathers (mean age, 42.8; SD, 4.6 years), and 579 mothers (mean age, 40.6; SD, 5.4 years). The number of fathers and mothers are less than that of children, because there were siblings among child participants. All respondents answered self-administered questionnaires via an Internet site or on a stand-alone personal computer. Participants provided their written informed consent if they agreed to participate in this survey. The full protocol for the J-SHINE data collection and informed consent procedure were approved by the internal Review Board at the University of Tokyo and the Ethics Committee of the Graduate School of Medicine at the University of Tokyo. Secondary data use for this study was approved by the J-SHINE Data Management Committee.

### Measurements

#### Children’s sedentary time

Our primary outcome was total sedentary time (min/day) during weekdays, and secondary outcomes were screen time (min/day) and non-screen time (min/day) on weekdays. Sedentary time was the sum of screen time and non-screen time. Screen time was calculated by summing up minutes per day of the following three activities; 1) TV/video/DVD, 2) PC and internet, and 3) games (computer game and TV game); and non-screen time was calculated by summing up minutes per day of the following two activities: 4) reading/music and 5) study. We also used these specific types of activities as the outcome variables. We obtained the self-report time of these activities from the following question: “Usually, how long do you do the following activity after school?; 1) Reading and listening to music, 2) Study (excluding study with a private tutor or in cram school), 3) Watching TV, video, or DVD, 4) PC and internet, 5) Game (computer game and TV game).” Children answered by providing minutes per day of each activity. Time of sedentary activities was rounded towards 99^th^ percentile of all children’s responded time; 900 min/day for sedentary time, 660 min/day for screen time, and 480 min/day for non-screen time.

#### Paternal and maternal working hours

The main explanatory variables were self-reported parental working hours from the question, “How many hours per week did you work on average over the past year? Please answer including overtime hours regardless of whether paid or unpaid.” Participants who answered “not working”, working “0 hour” and those who were on more than 13 months leave were categorized as not working (0 hour/week).

#### Covariates

We also investigated the following sociodemographic and behavioral factors as potential confounders: children’s age, children’s sex, number of siblings,^37^ child’s exercise time (min/week, via single question as following: “How many times (sessions) do you exercise a week? Also, how many hours a week do you exercise in total?”), parents’ age, parents’ educational attainment (categorized as <13, 13–15, or ≥16 years), equivalent household income calculated by dividing household annual income by the square root of the number of household members (categorized as low, middle, or high. Above 50^th^ percentile was high, 25^th^ to 50^th^ percentile was middle, and 0 to 25^th^ percentile was low).

### Statistical analysis

To investigate the association between children’s sedentary time and parental working hours, we conducted multiple linear regression analyses with robust error estimation adjusting for potential confounders. Model 1 adjusted for children’s age, children’s sex, number of siblings and children’s exercise time. Model 2 further adjusted for parents’ age, parents’ educational attainment, equivalent household income, and another parent’s working hours (maternal time for paternal time and vice versa). We categorized parental working hours into three groups using the median values among those who worked (ie, not working as a reference category, <48 hours/week, and ≥48 hours/week for fathers; and not working, <20 hours/week, and ≥20 hours/week for mothers). We tested for linear trends using continuous variables of maternal or paternal working hours. We conducted sub-group analysis, stratifying the child participants into elementary school children (aged 7–12 years) and junior high and high school children (aged 13–18 years). To investigate the possible mechanisms underlying the association between parental working hours and children’s sedentary behavior we also observed interactions between 1) paternal and maternal working hours and 2) parental working hours and socioeconomic factors (household income and parents’ educational attainments). To examine the interaction between paternal and maternal working hours, we used a combined category of maternal and paternal working hours as well. In addition, we conducted sensitivity analyses, 1) which excluded children aged under 10, since the validity of the measurement of sedentary behavior might be lower among those younger ages, 2) which additionally adjusted for nuclear family (yes or no), 3) which were stratified by nuclear family or not, and 4) which excluded children with fathers who did not work when analyzing maternal working hours as a main explanatory variable. All analyses were conducted using STATA (version 15.0, Stat Corp, College Station, TX, USA).

## RESULTS

There were 510 children aged 7–12 and 376 adolescents aged 13–18 (Table 1). On weekdays after school, children spent twice as long on sedentary screen time than non-screen time. There were 15 non-working fathers and 394 non-working mothers.

**Table 1.** Demographic characteristics of participants (*n* = 886)

		Mean (SD) or *n* (%)
Sex, boy		473 (53.4)
Age, mean (SD)		11.9 (3.3)
7–12		510 (57.6)
13–18		376 (42.4)
Number of siblings		
0		121 (13.7)
1		501 (56.5)
≥2		264 (29.8)
BMI, kg/m^2^, mean (SD)		17.6 (2.8)
Exercise time, min/week		314 (430)
Parents’ educational attainment,^a^ year		
Father,	≤12	233 (26.3)
	13–15	73 (8.2)
	≥16	580 (65.5)
Mother,	≤12	247 (27.9)
	13–15	412 (46.5)
	≥16	227 (25.6)
Equivalent household income,^b^ JPY/year		
Low (<2,795,085)		222 (25.1)
Middle (2,795,085–3,913,119)		276 (31.1)
High (>3,913,119)		388 (43.8)
Parental working hours,^a^ hours/week		
Father, mean (SD)		41.3 (23.2)
0		15 (1.7)
1–47		435 (49.1)
≥48		436 (49.2)
Mother, mean (SD)		12.6 (15.6)
0		394 (44.5)
1–19		215 (24.2)
≥20		277 (31.3)
Sedentary behavior on weekdays, min/day		
Total sedentary time		222 (123)
Screen time		144 (108)
TV/Video/DVD		89 (72)
Game		33 (46)
Internet		23 (41)
Non-Screen time		78 (65)
Reading/Music		27 (36)
Study		51 (51)

BMI, body mass index; SD, standard deviation.

^a^Parents were counted several times if they have multiple children.

^b^Calculated by dividing household annual income by the square root of the number of household members.

**Figure 1.**
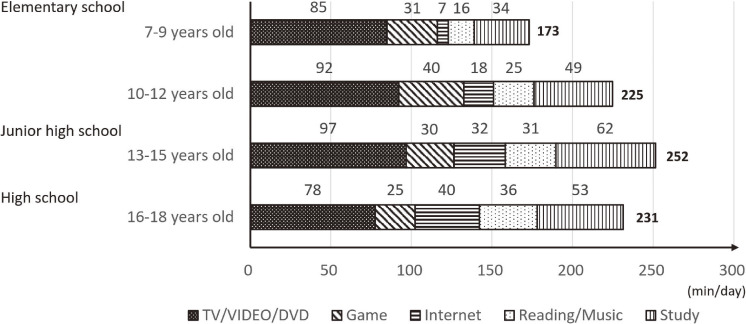
Average sedentary time broken down by types of activities children (*n* = 886) engaged in

Sedentary time during weekdays was the longest among children aged 13–15 who spent the most time watching TV/video/DVD (Figure 1). In all age groups, screen time comprised the largest proportion of the sedentary time. Nearly 43% of children aged 7–12, and half of adolescents aged 13–18 years, exceeded the recommended screen time of two hours/day. In addition, 28% of children aged 7–12 and 43% of adolescents aged 13–18 had sedentary time over 4 hours/day during weekdays (eFigure 1).

**Figure 2.**
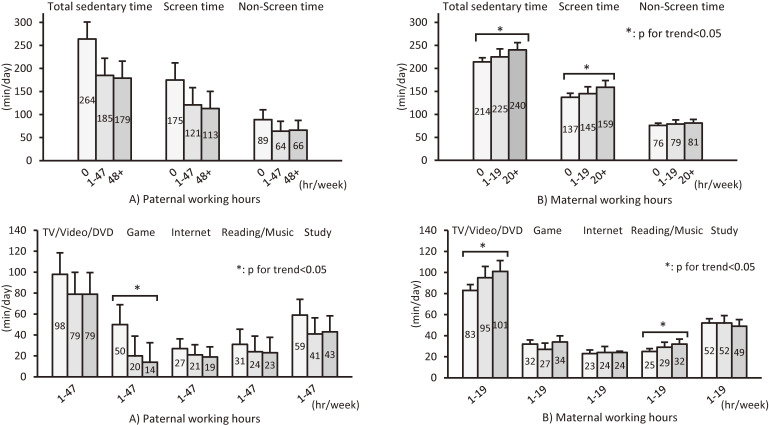
Adjusted predicted value and 95% CI of total sedentary time, screen time, non-screen time and specific sedentary activity of children by A) paternal and B) maternal working hours (*n* = 886). Data are adjusted for sex and age, number of siblings, exercise time (min/week), parents’ age, parents’ educational attainment, household income and another parent’s working hours (maternal time for paternal time and vice versa). CI, confidence interval.

In multiple regression analysis, even after adjusting for demographic and socioeconomic factors, children whose mothers worked longer than the median working hours had 28 min/day (95% confidence interval [CI], 9 to 48) longer sedentary time, especially TV/video/DVD viewing (18; 95% CI, 6 to 30 min/day), than children whose mothers were homemakers (Table 2 and Figure 2). The longer maternal working hours, the longer TV/video/DVD, reading/music, screen time, and sedentary time (*P* for trend ≤0.02). Clearer trends were observed among children aged 7–12 than those aged 13–18 (eFigure 2 and eFigure 3). In an analysis of sedentary time in all children, the coefficient of exercise time was −0.02 (95% CI, −0.04 to −0.002).

**Table 2.** Association between parental working hours (hours/week) and children’s total sedentary time, screen time and non-screen time on weekdays (min/day)

		Total sedentary time				Screen time		Non-Screen time	

		Model 1^a^		Model 2^b^		Model 2^b^		Model 2^b^	

		Coef. (95% CI)	*P* for trend	Coef. (95% CI)	*P* for trend	Coef. (95% CI)	*P* for trend	Coef. (95% CI)	*P* for trend
All (*n* = 886)									
Parental working hours									
Father	0	Ref.	0.14	Ref.	0.22	Ref.	0.14	Ref.	0.88
	1–47	**−76 (−151, −2)**		**−73 (−145, −1)**		−50 (−124, 23)		−23 (−64, 19)	
	≥48	**−82 (−156, −7)**		**−77 (−149, −5)**		−57 (−130, 16)		−20 (−62, 21)	
Mother	0	Ref.	**<0.01**	Ref.	**<0.01**	Ref.	**0.02**	Ref.	0.37
	1–19	12 (−8, 32)		11 (−9, 31)		8 (−9, 26)		2 (−8, 12)	
	≥20	**28 (9, 48)**		**26 (7, 46)**		**22 (4, 40)**		4 (−5, 14)	

Children aged 7–12 years (*n* = 510)									
Parental working hours									
Father	0	Ref.	0.18	Ref.	0.21	Ref.	0.11	Ref.	0.52
	1–48	−64 (−161, 34)		−67 (−161, 28)		−97 (−200, 6)		**31 (0, 61)**	
	≥49	−72 (−171, 26)		−74 (−169, 21)		**−104 (−207, −1)**		30 (−1, 61)	
Mother	0	Ref.	**<0.01**	Ref.	**<0.01**	Ref.	**<0.01**	Ref.	0.39
	1–19	18 (−7, 42)		19 (−5, 44)		19 (−3, 41)		1 (−9, 10)	
	≥20	**42 (15, 69)**		**39 (11, 66)**		**34 (9, 59)**		5 (−6, 16)	

Children aged 13–18 years (*n* = 376)									
Parental working hours									
Father	0	Ref.	0.77	Ref.	0.99	Ref.	0.97	Ref.	0.98
	1–45	−86 (−201, 29)		−77 (−193, 38)		20 (−56, 96)		**−97 (−162, −32)**	
	≥46	−83 (−197, 32)		−71 (−188, 46)		18 (−59, 94)		**−88 (−154, −23)**	
Mother	0	Ref.	0.96	Ref.	0.94	Ref.	0.88	Ref.	0.91
	1–21	0 (−30, 30)		2 (−28, 32)		−13 (−39, 14)		15 (−5, 34)	
	≥22	1 (−30, 32)		1 (−30, 33)		2 (−26, 30)		−1 (−18, 17)	

CI, confidence interval.

Boldface indicates *P* < 0.05.

^a^Model 1 adjusted for children’s sex and age, number of siblings, and children’s exercise time.

^b^Model 2 adjusted for covariates in model 1 plus parents’ age, parents’ educational attainment, equivalent household income and another parent’s working hours (maternal time for paternal time and vice versa).

On the other hand, after adjusting for potential confounders, children whose fathers worked longer had 82 (95% CI, −156 to −7) min/day shorter sedentary time than children whose fathers did not work (Table 2). Children whose fathers did not work spent 3.5 times (50 min/day vs 14 min/day) longer on games and 1.5 times (264 min/day vs 179 min/day) longer on sedentary time compared with children whose fathers worked longer hours (Figure 2). However, when limited to children whose fathers worked, there was no statistically significant association between any type of children’s sedentary behavior and paternal working hours. These trends were almost similar in terms of sedentary time between children aged 7–12 and those aged 13–18, although younger children did have these trends in screen time and older children in non-screen time (eFigure 2 and eFigure 3).

**Figure 3.**
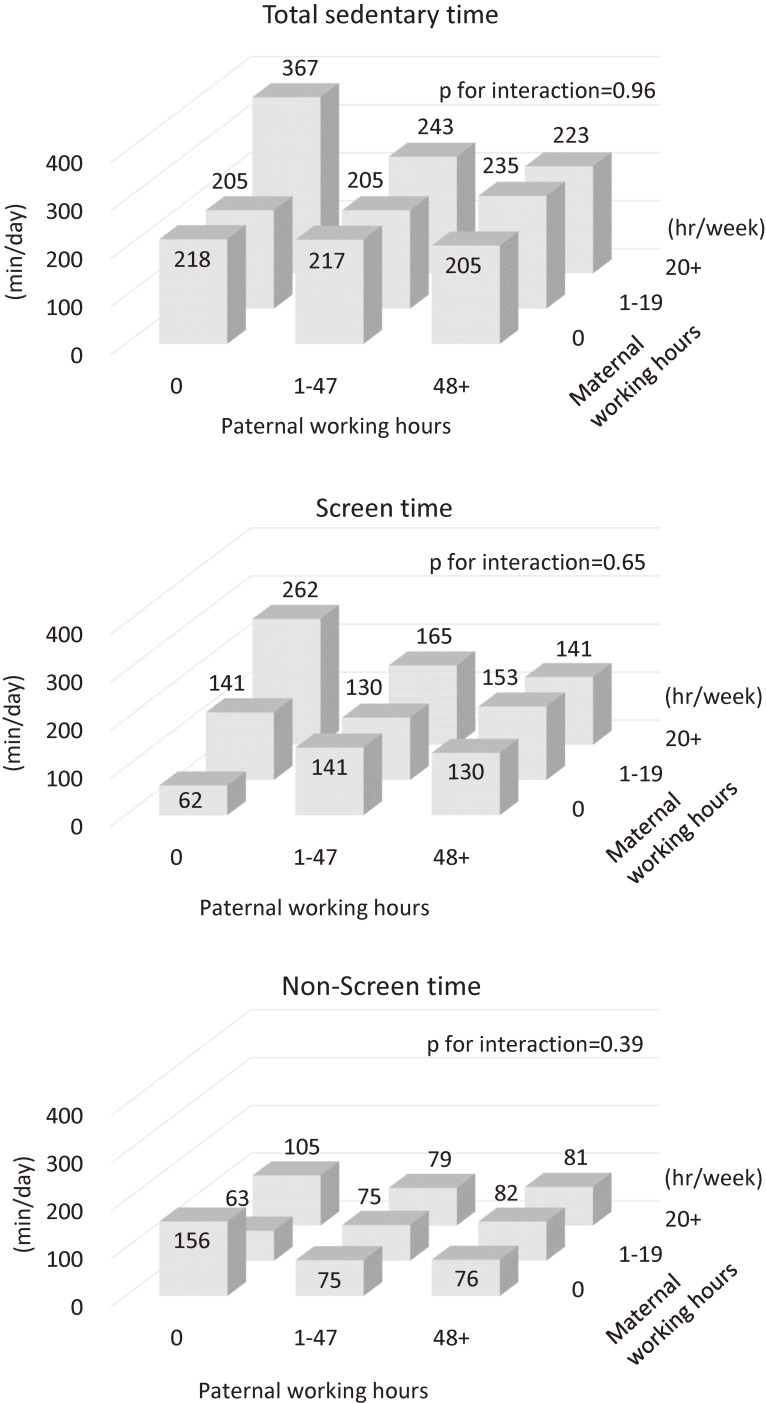
Interaction between maternal working hours and paternal working hours (*n* = 886)

Although the interaction between paternal and maternal working hours was not statistically significant (*P* ≥ 0.39), children with mothers who worked longer hours and fathers who did not work had 1.8 times longer sedentary time, compared with children whose mothers were homemakers and fathers worked more hours (367 min/day vs 205 min/day) (Figure 3). Interactions between paternal working hours and household income were statistically significant for screen time and sedentary time (*P* for interaction <0.01) (eFigure 4). Among children with low household income, sedentary time and screen time of children whose fathers did not work were longer than children whose fathers worked longer. In contrast, among children with high household income, those associations were inverse; sedentary times of children whose fathers did not work were slightly shorter than children whose fathers worked longer. Interactions between maternal working hours and household income and those between parental working hours and their educational attainment were not statistically significant (*P* for interaction ≥0.34) (eFigure 5, eFigure 6, and eFigure 7).

The results of the sensitivity analyses were similar to the main analyses. For example, in the sensitivity analysis which excluded children aged under 10, children whose mothers worked more than 20 hours had 18 (95% CI, −6 to 44) min/day longer sedentary time than children whose mothers were homemakers; children whose fathers worked longer had 91 (95% CI, −168 to −14) min/day shorter sedentary time than children whose fathers did not work.

## DISCUSSION

To our best knowledge, this is the first study to investigate the association between both paternal and maternal working hours and children’s sedentary time, which includes screen and non-screen time. This cross-sectional study found paternal and maternal working hours were differentially associated with children’s sedentary time during weekdays. Children whose mothers worked longer had more sedentary time than their counterparts due to longer screen time, especially TV viewing. Thus, as mothers worked longer, children tended to sit more. Conversely, children whose fathers did not work had more sedentary time than children whose fathers worked, yet no significant difference in children’s sedentary time was observed when limited to children whose fathers worked. These findings may suggest that among families that are deviated from typical traditional working conditions (ie, working father and homemaking mother), children tended to sit more time probably due to shortages in resources the families have, as discussed later in detail.

The average self-reported sedentary time during weekdays (222 min/day) in this study was within the range of those observed in previous studies.^18^^,^^38^^,^^39^ Prolonged screen time and sedentary time were also prevalent in children. Sedentary time was longer in adolescents than in elementary school children. These findings are consistent with recent data from other countries.^1^^,^^16^^,^^40^ Both Crepinsek and Burstein^27^ and Fertig et al^26^^,^^27^ showed that maternal employment status was associated with longer TV viewing among children, and children’s TV viewing increased as maternal working hours increased. Yamada et al^28^ also suggested that children whose mothers were full-time workers had prolonged screen time consisting of TV/DVD, Internet use, and games. Our study shows a comprehensive picture of the association between both paternal and maternal working hours and children’s sedentary time consisting of different types of activities.

As one of the possible mechanisms for the association between maternal working hours and children’s sedentariness, labor market participation among mothers produced time constraints and reduced the amount of time they spent on childcare.^25^ A previous study indicated childcare time reduced children’s TV viewing,^41^ this suggests that a shorter time of parental supervision might increase children’s indoor passive sedentary activities (eg, watching TV alone), rather than playing outdoors. Increased hours of paid work would improve financial resources and could be invested in their children; however, Hofferth and Sandberg^42^ suggested that children with non-working mothers rather tended to participate in activities, such as sports programs. Time constraints due to more work hours can inhibit those activities, for instance, parents cannot transport children to those clubs. Another possible mechanism, working mothers may sit and rest together with their children or they would let children watch TV while they perform housework instead of doing other interactive activities after work, as a result of mental distress^43^^,^^44^ or time pressure.^43^

Very few studies have examined the association between paternal working hours and children’s sedentary time. Similar to our findings, Hesketh et al^45^ indicated children whose fathers were not employed tended to have longer TV viewing time. In our study, younger children engaged in more gaming and TV viewing if fathers did not work, especially among families with lower household income. Paternal psychosocial stresses and difficulties due to unemployment might be a barrier preventing them from being involved in home discipline or caring for their children, such as playing with them outside. Since social norms and systems still have parts which have not become diversified, families deviating from typical traditional working conditions might suffer disadvantages. Future research using larger sample sizes or the other populations is needed to reveal whether maternal and paternal influences are independent of each other or there is some interplay.

There are countermeasures for parents and children to reduce screen time; for example, rule setting for TV viewing,^16^^,^^17^^,^^19^ reducing TV co-viewing,^18^^,^^19^^,^^46^ not setting up a TV in children’s bedrooms,^18^^,^^19^ better modeling for screen-based behavior by parents^17^^–^^19^^,^^28^ and parental involvement in sports^47^^,^^48^ or other activities.^18^ On the other hand, structural changes in the workforce, such as increased paid work for women, should be considered because it may be difficult for parents to reallocate time. There are potential health benefits for children by improving working conditions and work-life balance for their parents. In Japan, 7–18 year-old children spend their time at school from about 8:30 to 15:00 every weekday; junior high and high school students (13–18 years old) spend less time at home because they often join club activities after school and go to cram schools. This might lead to the current results, in which parental working hours were less likely to affect sedentary time at home for older (13–18 years old) children. The support for younger children and their parents is especially needed because younger children depend more on their household conditions. At present, there have been several measures by national and local governments intended to create places for younger children, such as after school children’s clubs and children’s cafeterias.^49^^,^^50^ However, in Japan, discussions on how children spend time after school from the perspective of reducing social inequity have just begun.^50^^,^^51^ Services for children’s after-school activities require significant improvement and supplement families’ resources by targeting the actual needs of the family. Our research could provide useful suggestions for researchers, people involved with children in educational or community settings, and policymakers to understand the importance of structural and social determinants of children’s lifestyles. Society- and community-wide systems and reformed norms for child care may help improving child health behavior. However, there is currently a dearth of evidence regarding the relationship between parental working style, childcare services, and children’s sedentary time. It is needed to investigate the effectiveness of policies and programs for reducing children’s sedentary behavior among families with various parental working conditions.

The strengths of this study include the investigation of both paternal and maternal working hours in addition to other sociodemographic factors, which allowed us to present a comprehensive picture of parental influence on children’s sedentary behavior. Moreover, we investigated various types of sedentary behavior consisting of screen time and non-screen time. However, our study has several limitations. First, since this study was a cross-sectional design, we are unable to address causality between outcomes and explanatory variables. Second, sedentary time and the other covariates (eg, exercise time) were collected using a self-report questionnaire. Although we measured similar items for sedentary time with validated questionnaires such as TV viewing, computer use, video games, and reading,^52^ we inquired regarding a limited domain (ie, leisure time on weekdays after school). Thus, we may have underestimated sedentary time as we did not include time spent at school or after-school club activities. We also did not assess how children spent their time on weekends or whether parents regulated their children, which may influence children’s weekdays’ time use. Wearable devices such as accelerometers can capture sedentary time per day objectively. It is possible that the validity was lower at younger ages. The distribution of sedentary behavior should be interpreted with care. However, the measurement error is considered to be non-differential to parental working status. It was an advantage to use a self-report questionnaire that enabled us to understand the behavioral context (ie, types) of sedentary behavior. Third, we analyzed only complete cases. We excluded participants from our analyses if they any had missing demographic, socioeconomic, or use of time data. If fathers who were not working tended not to answer those questions for some psychosocial reason and their children had longer sedentary time, our research may have underestimated the association. Internal validity should be considered with caution. Fourth, the external validity of the current results for the entire Japanese population is questionable, given that the J-SHINE participants are not nationally representative. Although the response rate is relatively low, it has been confirmed that the distribution of the demographic characteristics of the J-SHINE participants is representative of the targeted municipality residents.^36^ Additionally, since the number of children with fathers not working was relatively small, further research is warranted. Finally, the actual contents of each activity were not explored. For example, children may turn on the TV while studying, or children may engage in e-learning and read e-books via the internet for their studies. We did not consider those situations.

### Conclusion

Children with mothers who worked longer or fathers who did not work had prolonged sedentary time. This may attributable to the shortages in financial, temporal, and psychosocial resources for childcare among those families. Since the diversity in working styles has been increasing, society-wide reforms in policies and norms toward modifying the current conditions of strong parental obligations for child care may be necessary. Future research should investigate what types of policies and programs are effective in reducing children’s sedentary behavior among families with diverse parental working conditions.

## APPENDIX A. SUPPLEMENTARY DATA

eFigure 1. Distributions of total sedentary time, screen time, and non-screen time by age group

eFigure 2. Adjusted values of total sedentary time, screen time, non-screen time and specific activities of children aged 7–12 years by A) paternal and B) maternal working hours (*n* = 510) Data are adjusted for sex and age, number of siblings, exercise time (min/week), parents’ age, parents’ educational attainment, household income and another parent’s working hours (maternal time for paternal time and vice versa).

eFigure 3. Adjusted values of total sedentary time, screen time, non-screen time and specific activities of children aged 13–18 years by A) paternal and B) maternal working hours (*n* = 376) Data are adjusted for sex and age, number of siblings, exercise time (min/week), parents’ age, parents’ educational attainment, household income and another parent’s working hours (maternal time for paternal time and vice versa).

eFigure 4. Interaction between paternal working hours and equivalent household income (*n* = 886)

eFigure 5. Interaction between maternal working hours and equivalent household income (*n* = 886)

eFigure 6. Interaction between paternal working hours and paternal educational attainment (*n* = 886) There was no child of non-working fathers with 13–15 years of educational attainment.

eFigure 7. Interaction between maternal working hours and maternal educational attainment (*n* = 886)
