# Supplementary material for: Parental Working Hours and Children’s Sedentary Time: A Cross-sectional Analysis of the J-SHINE
Source: J Epidemiol. 2022 Jan 5;32(1):4–11. doi: 10.2188/jea.JE20200170 (PMC8666312; doi:10.2188/jea.JE20200170)
Supplement: Supplementary file 1 [file je-32-004-s001.pdf]

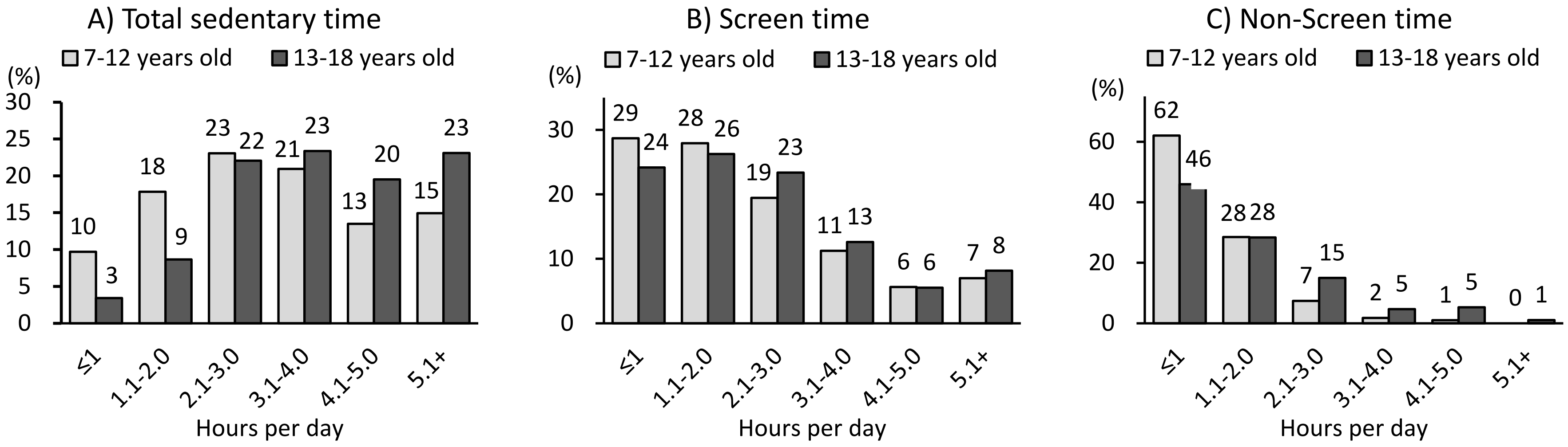

eFigure 1. Distributions of total sedentary time, screen time, and non-screen time by age group

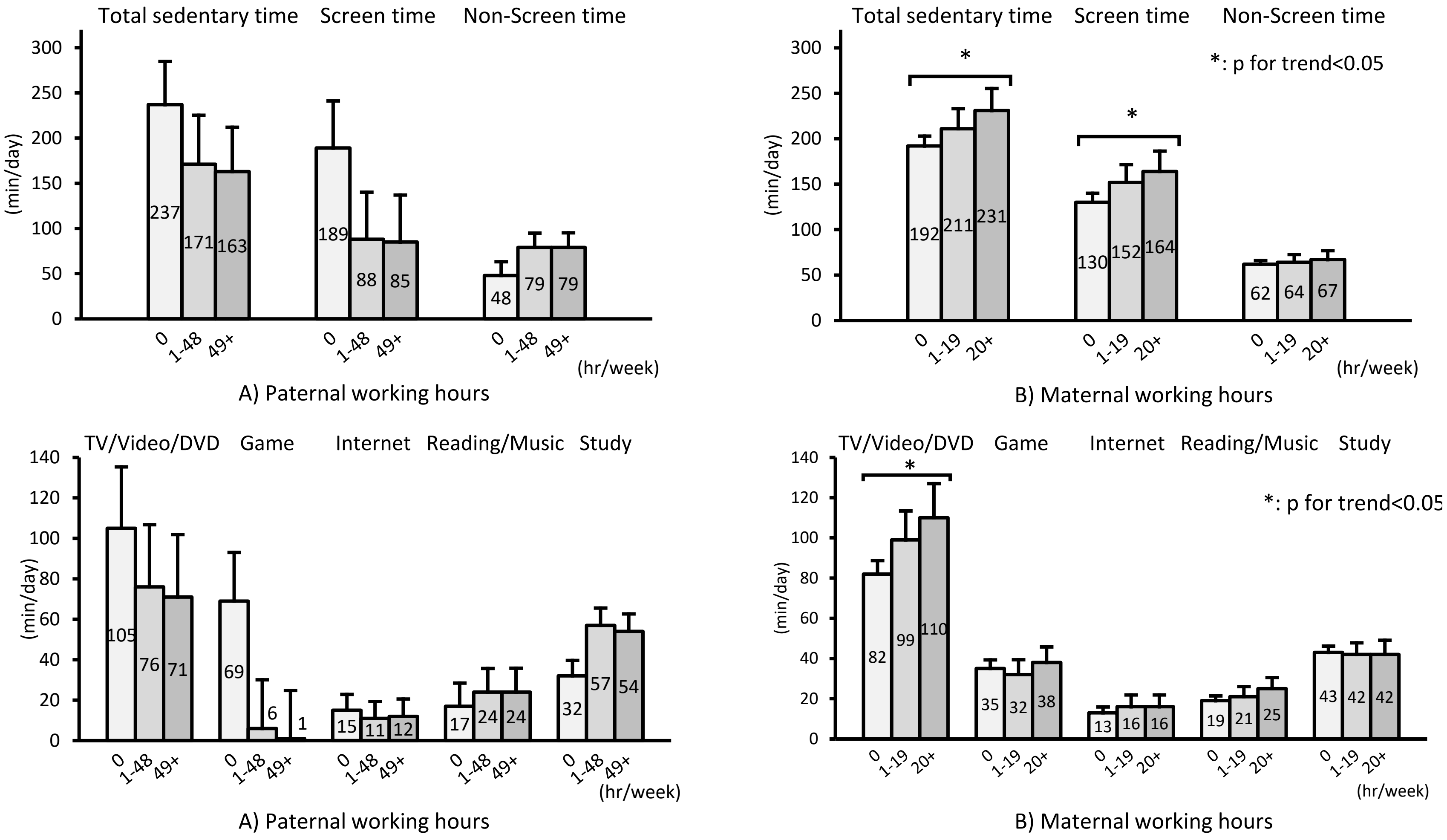

eFigure 2. Adjusted values of total sedentary time, screen time, non-screen time and specific activities of children aged 7-12 years by A) paternal and B) maternal working hours (n=510)
Data are adjusted for sex and age, number of siblings, exercise time (min/week), parents' age, parents' educational attainment, household income and another parent's working hours (maternal time for paternal time and vice versa).

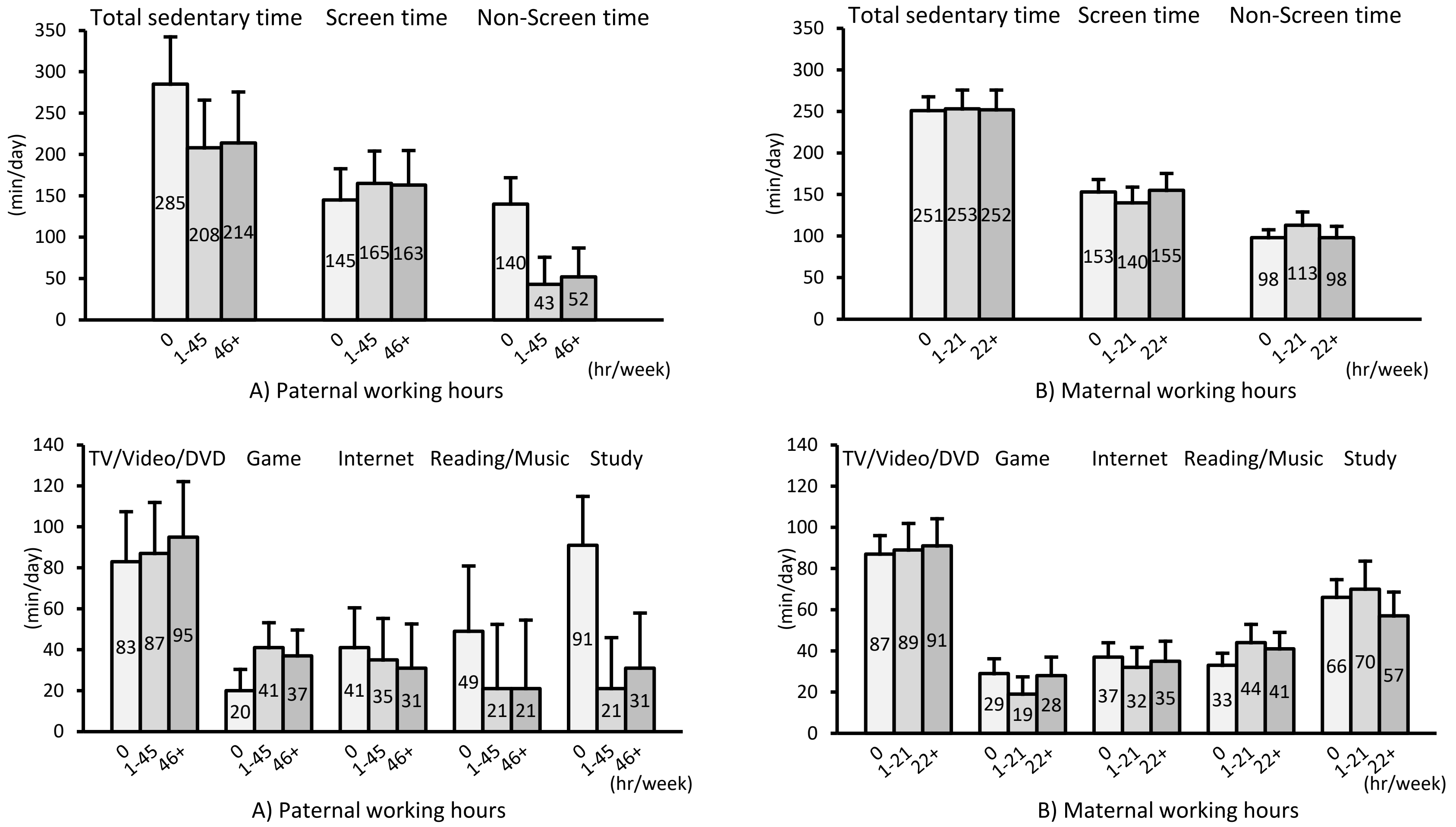

eFigure 3. Adjusted values of total sedentary time, screen time, non-screen time and specific activities of children aged 13-18 years by A) paternal and B) maternal working hours (n=376)
Data are adjusted for sex and age, number of siblings, exercise time (min/week), parents' age, parents' educational attainment, household income and another parent's working hours (maternal time for paternal time and vice versa).

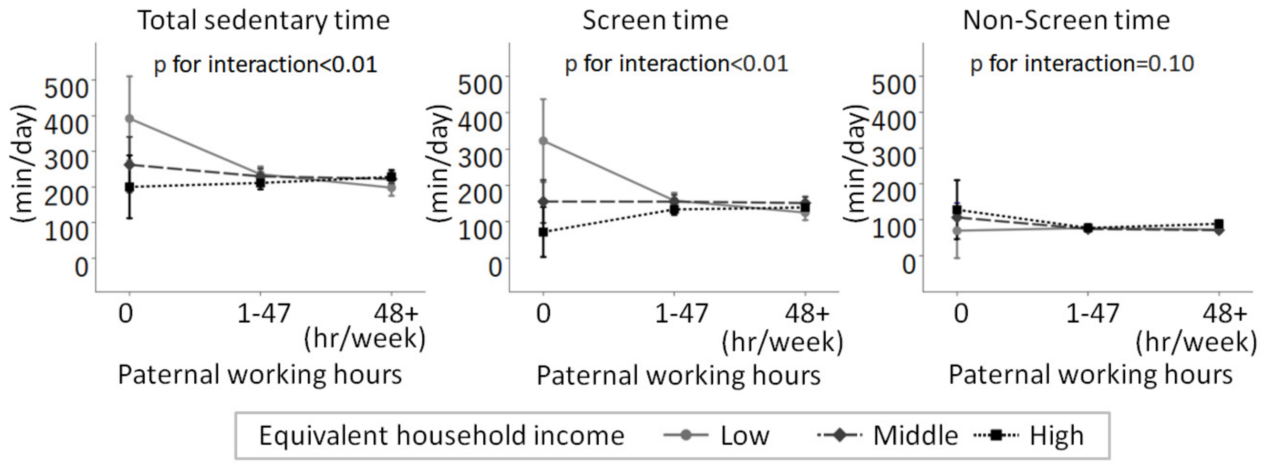

eFigure 4. Interaction between paternal working hours and equivalent household income (n=886)

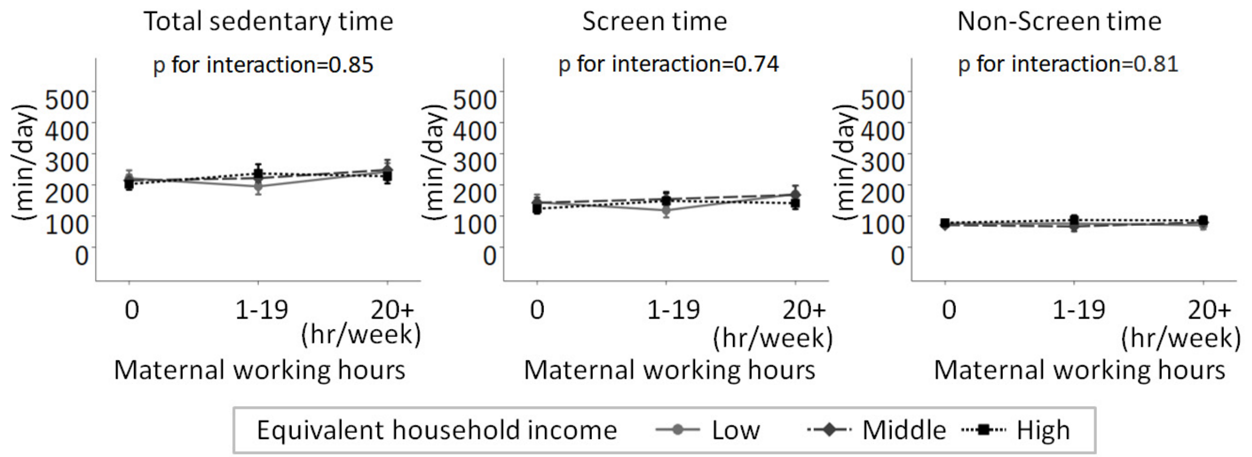

eFigure 5. Interaction between maternal working hours and equivalent household income (n=886)

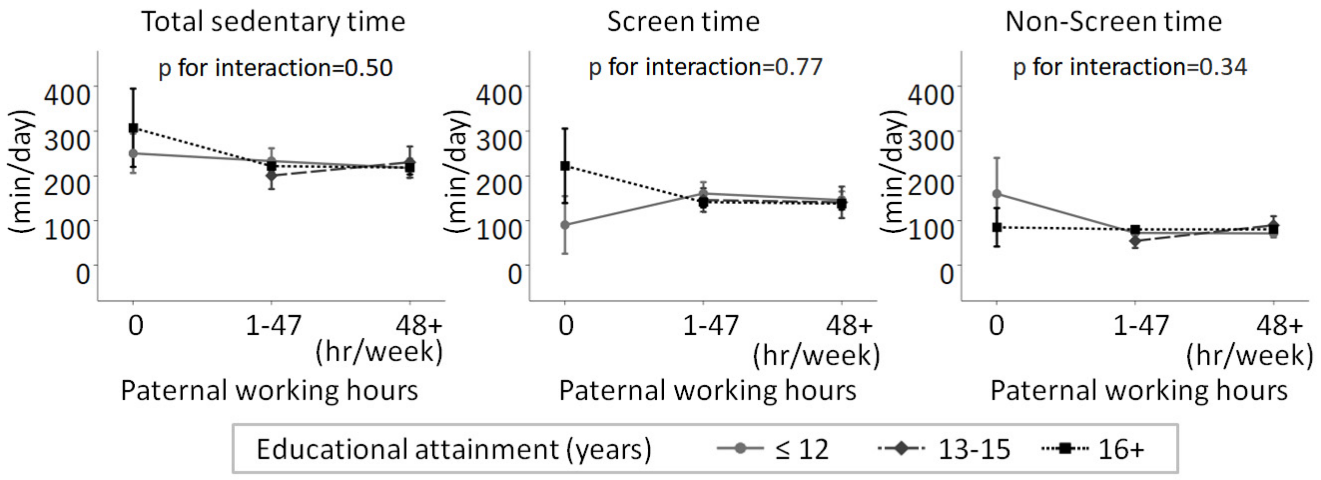

eFigure 6. Interaction between paternal working hours and paternal educational attainment (n=886)
There was no child of non-working fathers with 13-15 years of educational attainment.

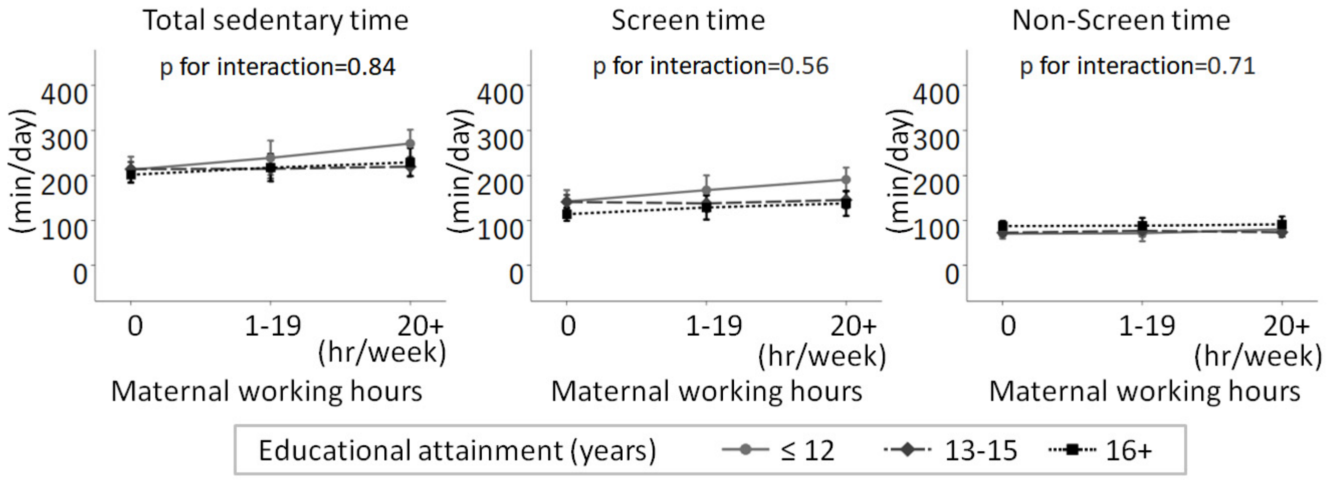

eFigure 7. Interaction between maternal working hours and maternal educational attainment (n=886)
